# DUF1005 Family Identification, Evolution Analysis in Plants, and Primary Root Elongation Regulation of *CiDUF1005* From *Caragana intermedia*

**DOI:** 10.3389/fgene.2022.807293

**Published:** 2022-03-29

**Authors:** Xiaona Tian, Xiaocui Niu, Ziru Chang, Xiujuan Zhang, Ruigang Wang, Qi Yang, Guojing Li

**Affiliations:** ^1^ Inner Mongolia Key Laboratory of Plant Stress Physiology and Molecular Biology, Inner Mongolia Agricultural University, Hohhot, China; ^2^ Key Laboratory of Forage Cultivation, Processing and High Efficient Utilization, Ministry of Agriculture, Inner Mongolia Agricultural University, Hohhot, China; ^3^ Key Laboratory of Grassland Resources, Ministry of Education, Inner Mongolia Agricultural University, Hohhot, China

**Keywords:** DUF1005, family identification, phylogenetic analysis, primary root, Caragana intermedia

## Abstract

Proteins with a domain of unknown function (DUF) represent a number of gene families that encode functionally uncharacterized proteins in eukaryotes. In particular, members of the DUF1005 family in plants have a 411-amino-acid conserved domain, and this family has not been described previously. In this study, a total of 302 high-confidence DUF1005 family members were identified from 58 plant species, and none were found in the four algae that were selected. Thus, this result showed that DUF1005s might belong to a kind of plant-specific gene family, and this family has not been evolutionarily expanded. Phylogenetic analysis showed that the DUF1005 family genes could be classified into four subgroups in 58 plant species. The earliest group to emerge was Group I, including a total of 100 gene sequences, and this group was present in almost all selected species spanning from mosses to seed plants. Group II and Group III, with 69 and 74 members, respectively, belong to angiosperms. Finally, with 59 members, Group IV was the last batch of genes to emerge, and this group is unique to dicotyledons. Expression pattern analysis of the CiDUF1005, a member of the DUF1005 family from Caragana intermedia, showed that CiDUF1005 genes were differentially regulated under various treatments. Compared to the wild type, transgenic lines with heterologous CiDUF1005 expression in Arabidopsis thaliana had longer primary roots and more lateral roots. These results expanded our knowledge of the evolution of the DUF1005 family in plants and will contribute to elucidating biological functions of the DUF1005 family in the future.

## Introduction

A domain with no confirmed function in the Pfam database is generally called a domain of unknown function (DUF). These domains have two distinct characteristics, including a relatively conserved amino acid sequence and an unknown function (Bateman et al., 2010). In total to date, the Pfam database (version 34) contains 19,179 families, including 6,565 DUF or UPF (uncharacterized protein families), which occupies approximately 34% of all the Pfam families (Mistry et al., 2020). Although the functions of some DUF families have been characterized, a large set of DUF members remains unknown.

Previously published studies have described the vital role of DUF domain-containing proteins in biotic stresses (such as insect pests), abiotic stresses (such as cold, heat, drought, and salt), and plant growth and development. Some DUFs were found to be involved in defense responses and endowed plants with a tolerance to pests and diseases, for example, *DUF26* (Wrzaczek et al., 2010; Miyakawa et al., 2014; Lee et al., 2017) and *DUF538* (Gholizadeh, 2011; Gholizadeh and Kohnehrouz, 2013; Gholizadeh, 2020). *AtRDUF1*, *AtRDUF2* (Kim et al., 2012; Li et al., 2013), *DUF1645* (Cui et al., 2016), and *DUF966* (Luo et al., 2014; Zhou et al., 2020) were demonstrated to have a role in the responses of plant to abiotic stress. *DUF266* (Parsons et al., 2012), *DUF231* (Yuan et al., 2013), *DUF246* (Oikawa et al., 2010), *DUF1218* (Mewalal et al., 2016), and *DUF579* (Urbanowicz et al., 2012) of *Arabidopsis thaliana* are commonly reported to regulate the development of plant cell walls. Several studies have confirmed that some DUFs, such as DUF640 (Zhao et al., 2004) and DUF827 (Kodama et al., 2011), affect chloroplast development and the growth of plants.

In addition to regulating the plant cell wall and chloroplast, DUF also regulates the development of plant roots, leaves, flowers, and fruits. JA-induced DUF26 functions in rice root development (Jiang et al., 2007), at the same time, and is related to the stomatal density (Arellano-Villagómez et al., 2021). The DUF724 family was highly expressed in *Arabidopsis* apical meristem tissues (shoots, leaves, roots, etc.) (Cao et al., 2009). In addition, there were many DUFs that regulated root development. These include DUF828 (Prabhakaran Mariyamma, 2016), DUF966 (Shen et al., 2019), DUF668 (Zhao et al., 2021), DUF642 (Gao et al., 2012; Salazar-Iribe et al., 2016), and DUF761 (Zhang et al., 2019). Sugar accumulation 1 (OsSAC1), which consists of two conserved DUFs, namely, DUF4220 and DUF594, causes sugar to accumulate in rice leaves (Zhu et al., 2018). DUF647 regulates the early development of roots through UV-B during root development (Leasure et al., 2009). While DUF640 family members were mainly involved in the regulation of flower development, the DUF784 and DUF1216 (Huang et al., 2008) families were found to play a role in pollen development (Jones-Rhoades et al., 2007).

DUF1005 domain-containing proteins are exclusively present in plant genomes. As of July 2021, a total of 937 genes belonging to the DUF1005 family (Pfam accession: 6219) could be retrieved from 138 species in the Pfam database. Previous studies have shown that *DUF1005*s were differentially expressed in the transcriptome of cotton, and it was associated with fiber uniformity and fiber elongation (Okubazghi et al., 2017). There were a total of 4 *DUF1005*s in cucumbers, and they were related to controlling the cucumber lengths (Zhang, 2019). Moreover, proteins with a DUF1005 domain were found in cell wall proteome studies, indicating that they might have functions in cell wall development (Sergeant et al., 2019). The DUF1005-containing protein stigma exposed 1 (SE1) from *Vigna radiata* has been reported to affect petal and pistil growth during the late development stages in the *se1* mutant (Yun et al., 2020). However, few studies have focused on the function of DUF1005 in *Caragana intermedia*.

*C. intermedia* is a xerophytic deciduous shrub that belongs to the genus *Caragana* Fabr. It is mainly distributed in the arid and semiarid desert areas of Inner Mongolia, Ningxia, Shanxi, and northern Shaanxi. It is a native desert shrub with strong drought, salinity, cold resistance, sand-fixing capacity, and high forage value and is a superior xerophytic shrub species that is highly suitable for artificial afforestation in arid desert steppes (Liu et al., 2019). In this study, we identified a gene in *C. intermedia* that encodes DUF1005, and the morphology and abiotic stress response of *CiDUF1005* were further studied in transgenic *Arabidopsis*. In addition, we performed an analysis of the DUF1005 family in 58 representative plant species, including the identification and evolutionary analysis of DUF1005 family members in 58 representative plant species. The results obtained here should broaden our understanding of the roles of the DUF1005 family and provide a framework for further functional investigation of these genes in plants.

## Materials and Methods

### Data Retrieval and Identification of DUF1005 Genes

We selected species that are representative of the various stages, based on the progression of plants from moss to plants with seeds in evolution. We identified DUF1005 candidate genes from 58 representative plant species. The protein databases were downloaded from Phytozome12 (Goodstein et al., 2011), ConGenIE (Sundell et al., 2015), and NCBI. Detailed genomic information for the 58 species included in this study is listed in Supplementary Table S1.

To identify the DUF1005 gene family members in the above species, a profile hidden Markov model (HMM) (pHMM) of the DUF1005 (Pfam: PF06219) domain was downloaded from Pfam 31.0 (Mistry et al., 2020). Hmmsearch (HMMer package version 3.1b1) was used to search DUF1005.hmm against the protein sequences from each plant genome (Eddy, 1998). To ensure that the search was reliable, domain hits beyond the gathering threshold (E-value 1e−10) were filtered out before downstream analysis. For gene loci with multiple predicted isoforms, the primary isoform was used if the primary isoform annotation was available; otherwise, the longest protein was used. The Pfam database was employed to confirm the DUF1005 domain in the candidate proteins. The number and completeness of DUF domains were queried on the Conserved Domain Database (CDD) (https://www.ncbi.nlm.nih.gov). According to the 303 protein sequence of DUF1005, we searched homologous proteins in 4 algae with homology comparison in blast+ (Camacho et al., 2009) with above 10^−5^ e-value.

The compute pI/MW, a tool of the ExPASy server (http://web.expasy.org) was used to calculate the molecular weight (MW) and theoretical isoelectric point (pI) of the DUF proteins (Wiederschain, 2006). The WoLF PSORT program (https://wolfpsort.hgc.jp/) was used to predict protein subcellular localization (Horton et al., 2007).

### Phylogenetic Analysis and Conserved Motif Analysis

The amino acid sequences of the DUF1005s identified from 58 plants were subjected to alignment by ClustalW with default settings (Larkin et al., 2007). Phylogenetic analysis was conducted using the maximum likelihood (ML) method. First, the ML estimation using the best-fitting model JTT+F+R6 of sequence evolution was determined by ModelFinder (Kalyaanamoorthy et al., 2017). Then, IQ-TREE (Trifinopoulos et al., 2016) was used to infer the ML tree with 1,000 bootstrap replicates for alignment (Nguyen et al., 2014). All phylogenetic trees were edited and displayed with the online tool iTOL (Letunic and Bork, 2006). MEME software v5.0.5 (http://memesuite.org/tools/meme) was employed to identify conserved motifs with the default parameters (except that the maximum number of motifs was set to 6) (Bailey et al., 2009; Chen et al., 2020). The map was redrawn with Tbtools (Chen et al., 2020).

### Cloning the Full-Length CiDUF1005 cDNA and Genomic DNA

Total DNA and RNA were isolated using a Plant Genomic DNA Kit (DP305) and RNAprep Pure Plant Kit (DP419) (Tiangen, Beijing, China). The genomic DNA was removed, and the total RNA was converted to cDNA using TransScript® One-Step gDNA Removal and cDNA Synthesis SuperMix (AT311-02) (TransGen, Beijing, China). We identified a sequence containing the DUF1005 domain from the drought-treated transcriptome database (SRA accession number: SRP121096) of *C. intermedia* and named it *CiDUF1005*. To clone the full-length cDNA and gDNA of *CiDUF1005* from *C. intermedia*, PCR was set up with 5× PrimeSTAR Buffer (Mg^2+^ Plus) (Takara, Dalian, China) of 10 μl, dNTP Mixture (2.5 mM each) of 4 μl, *CiDUF1005*HA-F/R at 1 μl each, PrimeSTAR HS DNA Polymerase (2.5 U/μl) of 0.5 μl, template cDNA/gDNA of 5 μl, and ddH_2_O supplemented up to 50 μl. Then, the thermocycler program consisted of 30 cycles at 98°C for 10 s, 70°C for 10 s, and 72°C for 3 min to amplify the target products. The genes were then cloned and sequenced. The GSDS website (Gene Structure Display Server, http://gsds.cbi.pku.edu.cn) was used to analyze the CiDUF1005 structure (Hu et al., 2014). Multiple sequence alignment was carried out using DNAMAN (6.0.3.99) software. Phylogenetic analysis was performed by using MEGA 7 (Kumar et al., 2016) software. The alignment was adjusted manually, while the unrooted phylogenetic trees were constructed by the neighbor-joining method (Sievers et al., 2011).

### Construction of the Expression Vector and Genetic Transformation

To produce a recombinant for the constitutive expression of *CiDUF1005*, *Spe*I and *Sac*I (Takara, Dalian, China) sites were introduced at the end of the full-length cDNA sequence of *CiDUF1005* by PCR amplification, and then, the fragment was digested and subcloned into the *pCanG-HA* vector (which was kindly provided by Prof. Qi Xie, Institute of Genetics and Developmental Biology, Chinese Academy of Sciences, China) that was driven by the CaMV35S promoter. The resulting recombinant plasmid was sequenced to confirm that there were no PCR errors. For the plant transformation, the *pCanG-HA-CiDUF1005* recombinant vector and empty vector were transferred into *Agrobacterium tumefaciens* strain GV3101 by electroporation. The *Agrobacterium*-mediated floral dip method was used for *Arabidopsis* transformation (Clough and Bent, 1998). Transgenic lines were first selected by kanamycin (25 mg/L), then a total of seven homozygous transgenic lines were obtained, and the expression of *CiDUF1005* was verified by qRT–PCR. At the same time, empty vector lines of *pCanG-HA* were obtained using the same method. Homozygous seeds from the transgenic lines were used for subsequent research. All primers used in this study are listed in Supplementary Table S5.

### Plant Materials and Growth Conditions

In this experiment, the *Arabidopsis* ecotype Col-0 (Columbia-0) was used as the wild type (WT) and for the genetic transformation of *CiDUF1005*. All seeds from the WT and transgenic lines were kept in darkness for 3 days at 4°C on 0.6% agar medium with half strength (1/2) Murashige and Skoog (MS) after the seeds were sterilized. The seeds were then sown on 1/2 MS medium containing 2.3 g/L of Murashige and Skoog Basal Medium with vitamins (PhytoTech, Lenexa, KS, USA), 1% (w/v) sucrose (Sigma, USA), 0.025% MES (Coolaber, Beijing, China), and 10 g/L of plant agar (Chembase, Nantong, China), and the pH was adjusted to 5.7 with KOH. Plates were cultured for 11 days under long-day conditions (16-h light/8-h dark cycle) at 22°C. Pictures were obtained each day from the first day to the 11th day that the primary root length was measured, and the relative elongation was calculated. Thirty-six seedlings were used to measure root length for each line, and three independent biological replicates were carried out.

### Quantitative Real-Time PCR

Seeds from *C. intermedia* were collected from Hohhot, Inner Mongolia, China. One-month-old seedlings that were sown in pots containing a soil mixture were used to detect the transcript level of *CiDUF1005* under various treatments. For the cold, heat, drought, dehydration, and ABA treatments, we referred to Dr. Xiaomin Han’s method (Han et al., 2015). Three independent biological replicates, each comprising three individual plants, were used for quantitative real-time PCR. Triplicate quantitative assays were performed with each cDNA sample.

For qRT–PCR analysis, the obtained cDNA was diluted with RNase-free water before use. The target genes were expressed with specific primers, and qRT–PCR was performed using SYBR Green I Master Mix (Roche, Basel, Switzerland) and a LightCycler 480 Real Time PCR system (Roche, Switzerland). The thermal cycling program was 95°C for 5 min, followed by 40 cycles of 95°C for 5 s and 60°C for 30 s. The melting curves were analyzed at 60°C–95°C after 40 cycles. The relative RNA transcript level was calculated according to the 2^−ΔΔCT^ method (Livak and Schmittgen, 2001). *AtEF1α* (*AT5G60390*) and *CiEF1α* [GenBank No.: KC679842] were used to normalize the *Arabidopsis* and *C. intermedia* samples, respectively (Yang et al., 2014). All primers were designed by Primer Premier 5.0 and are listed in Supplementary Table S5. All qRT–PCR assays were carried out with three technical replicates.

### Statistical Analysis

The data in this research were subjected to statistical analysis using Microsoft Excel 2016 and SPSS. All data are presented as the mean of three biological replicates ±SD. A statistical analysis was carried out using at least 36 seedlings in each phenotypic experiment. The significant difference between groups was analyzed by Student’s t-test, Duncan’s multiple range tests, and one-way ANOVA. Significant differences were evaluated at *p* < 0.01 or *p* < 0.05.

## Results

### Identification of the DUF1005 Gene Family in Plants

**FIGURE 1:**
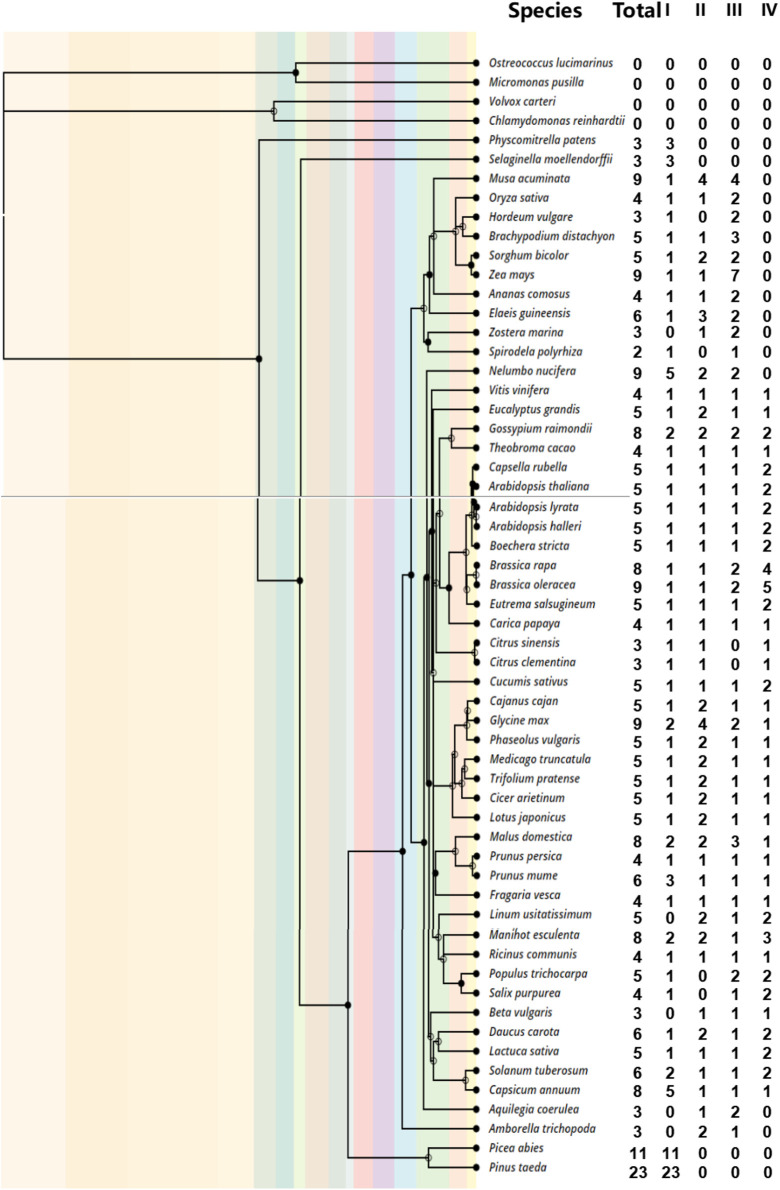
The phylogeny of the 58 plants analyzed in this study and the number of *DUF1005* genes identified. The order of tree branches and divergence time was derived from the TimeTree database (http://timetree.org/).

To investigate the number of DUF1005 family members in different species, we conducted a search for DUF1005 across plant lineages, including 58 representative species, by an HMM search. Information on the species and genomes used here is listed in Supplementary Table S1. In the end, 302 full-length DUF1005 were identified from 54 plant species, including the four major plant lineages of bryophytes, lycophytes, angiosperms, and gymnosperms (Figure 1). Then, the protein database was blasted by using the query sequences of the DUF1005 family in 54 plant species and four algae species. The E-value cutoff was set at 1.0e−5 to ensure confidence. We further dissected the algae (*Volvox carteri*, *Chlamydomonas reinhardtii*, *Ostreococcus lucimarinus*, and *Micromonas pusilla*), and no DUF1005 was found.

According to the number of genes analysis (Figure 1), we found that *Spirodela polyrhiza* harbored only two DUF1005s, and it ranked last among 54 plant species. In contrast, two gymnosperms, *Pinus taeda* and *P. abies*, presented the most DUF1005 members, with 23 and 11, respectively.

The number of DUF1005 family members was relatively small in all plants. Among the other 53 angiosperms except for *S. polyrhiza*, the number of DUF1005 genes ranged from 3 to 9. For example, there were 3 DUF1005 sequences in 9 species including *Physcomitrella patens*. Another 9 species, including *Oryza sativa*, contain 4 genes. There were 6 sequences in 4 species, including *Fragaria vesca*. There were 8 sequences in 5 species, including *Gossypium raimondii*. There were 9 sequences in 5 species, including *Zea mays*. Nineteen species, such as *Lotus japonicus*, contained 5 DUF1005 sequences in their genomes. Overall, there were few changes in the number of *DUF1005* genes among the different plants.

For further verification or detection of the number and completeness of DUF1005 structural domains, we screened out the DUF1005 domain in the NCBI conserved structural domain database (Supplementary Table S2). Most of the identified *DUF1005* gene-coding proteins only contained one DUF1005 domain; nevertheless, there were some exceptions. For example, MA_178396g0010 (*P. abies*) included two deleted DUF1005 domains, PITA_000036082-RA (*P. taeda*) contained a complete DUF1005 domain and a missing domain, and GSMUA_Achr4P08250_001 (*Musa acuminata*) contained a complete DUF1005 domain and a PTH2_family_superfamily domain. In addition to the three examples shown above, there were also other proteins with deletions in DUF1005 domains. Among them, the C-terminal domain was deleted in 15 proteins, the N-terminal domain was deleted in 22 proteins, and both ends were deleted in 8 proteins.

### Phylogenetic Analysis of the DUF1005 Gene Plant Family

**FIGURE 2:**
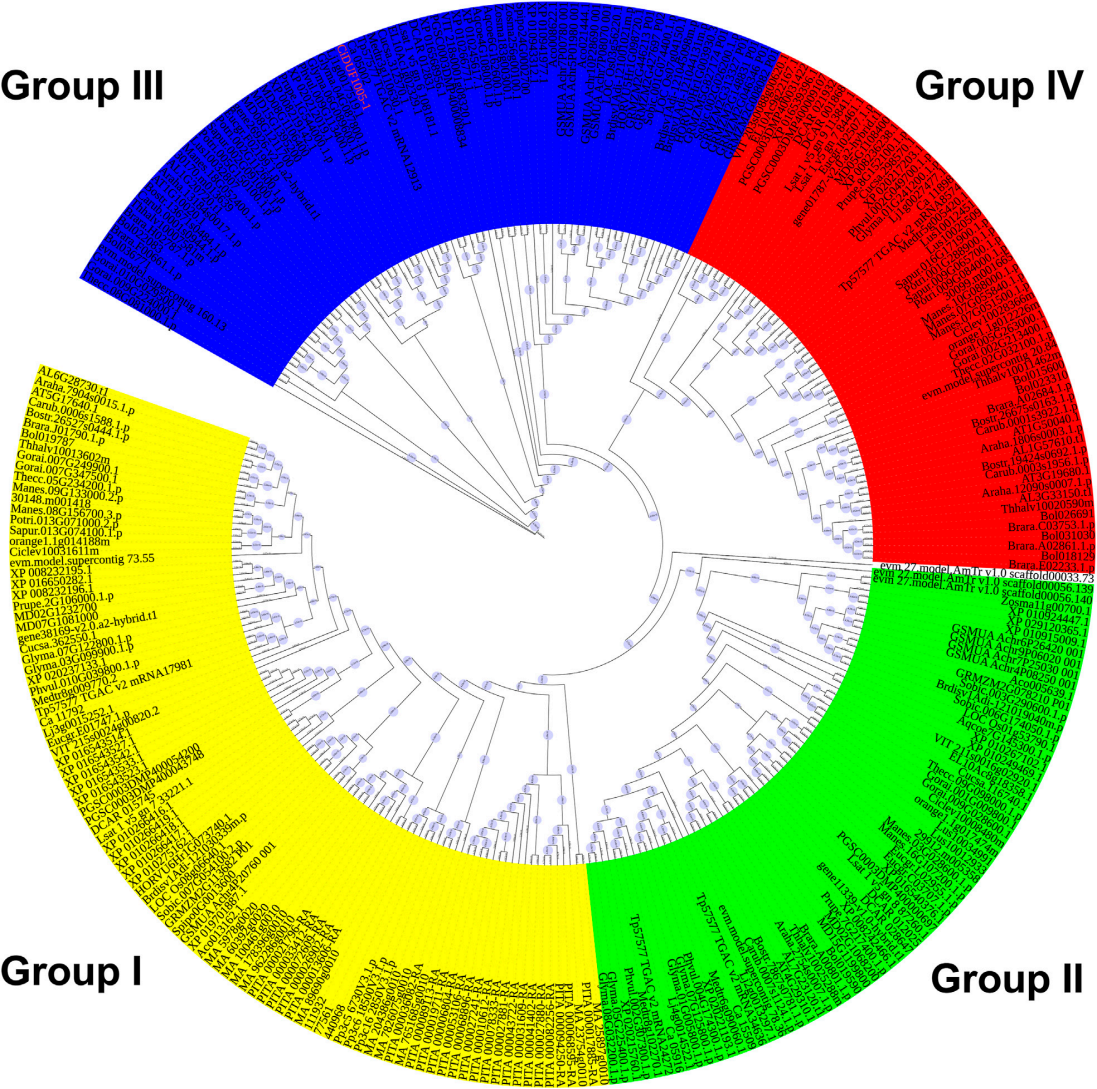
Phylogenetic classification of *DUF1005* genes in plants. A phylogenetic tree was constructed using the maximum likelihood (ML) method with IQ-tree. The yellow color represents Group I, the green color represents Group II, the blue color represents Group III, and the red color represents Group IV.

To explore the evolutionary relationship of the DUF1005 family in plants, a phylogenetic tree was constructed. IQ-tree software was used to perform a systematic evolutionary analysis of the 302 proteins identified and CiDUF1005 of *C. intermedia* by the ML method (Figure 2 and Supplementary Figure S1). It is clear that the DUF1005 plant family is divided into four subfamilies, namely, Group I, Group II, Group III, and Group IV (Figure 2 and Supplementary Table S3). The results showed that Group I included a total of 100 genes. Group II and Group III had 69 and 74 genes, respectively. The last group, marked as Group IV, contained 59 genes. The group that emerged the earliest was Group I, and the members of Group I was widely distributed from bryophytes (mosses) to angiosperms (seed plants) except *Beta vulgaris*, *Linum usitatissimum*, *Aquilegia coerulea*, *Zostera marina*, *Amborella trichopoda*, and the four algae. Moreover, Group I was ubiquitously distributed in all species, from mosses to seed plants, while Group II and Group III were only found in most angiosperms. The DUF1005 in Group II existed in most angiosperms except *G. raimondii*, *Salix purpurea*, *Hordeum vulgare*, and *S. polyrhiza*. The DUF1005 of Group III also existed in most angiosperms except *Citrus sinensis* and *Citrus clementina*. Obviously, Group IV was the last to emerge, and it is a specific group to dicotyledons in addition to *A. coerulea* and *Nelumbo nucifera*. The CiDUF1005 of *C. intermedia* used in this report belonged to Group III. This implied that the divisions of the four subgroups occur after the gymnosperms to angiosperms.

### Analysis of DUF1005 Protein Features

**FIGURE 3:**
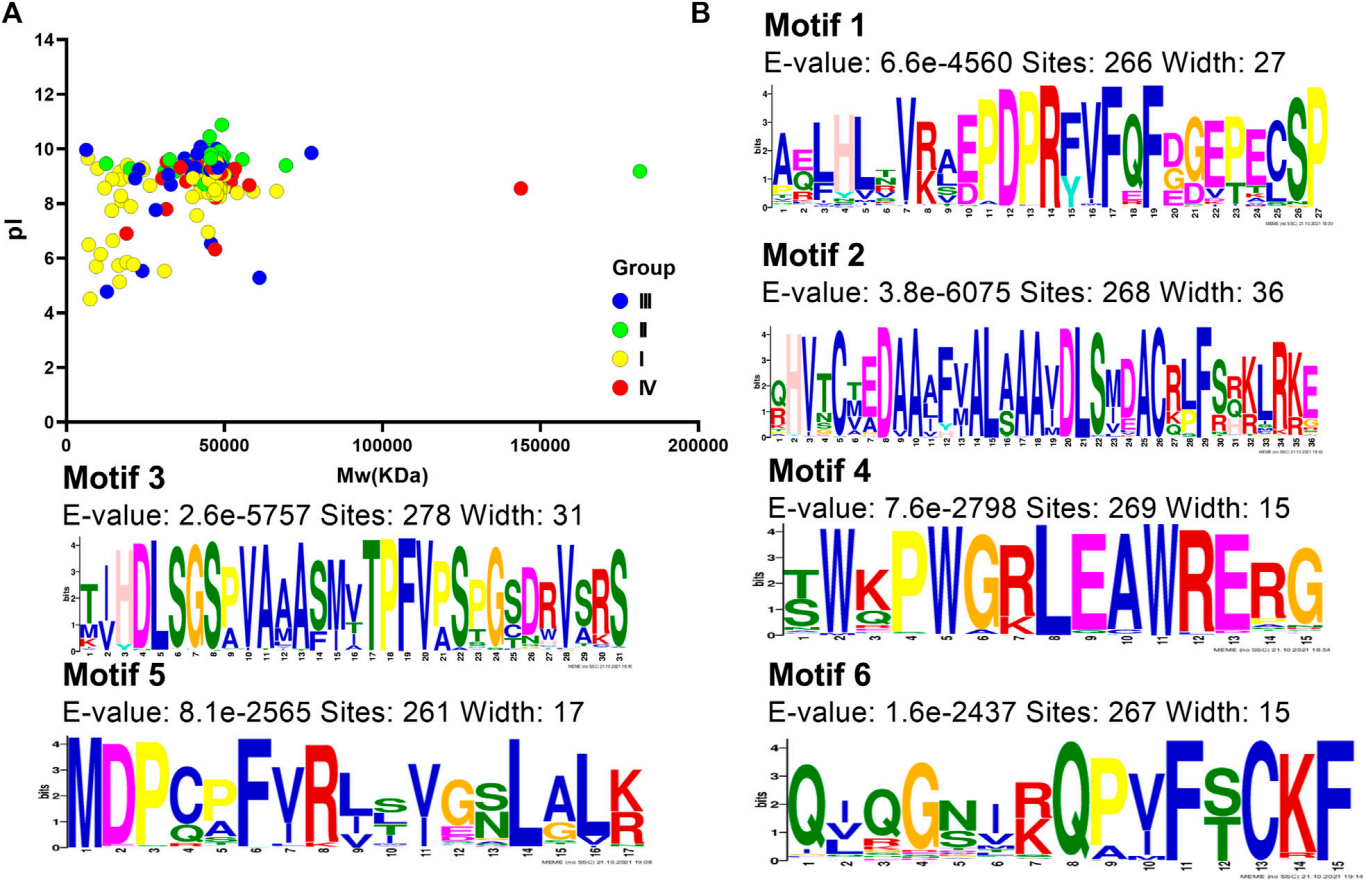
DUF1005 protein features. **(A)** Molecular weight (Mw) and isoelectric point (pI) of *DUF1005* genes in plants. The yellow color represents Group I, the green color represents Group II, the red color represents Group III, and the blue color represents Group IV. **(B)** Sequence logos for the conserved motifs of DUF1005 proteins in plants.

Furthermore, we analyzed the protein characteristics of these 303 DUF1005 (including CiDUF1005) sequences. The results showed that DUF1005 proteins varied greatly from 59 amino acids (aa) to 1,656 aa, with an average length of 411 aa (59 aa–1656 aa). All of the *DUF1005* candidates and their encoded protein features are listed in Supplementary Table S4. The features of DUF1005 proteins were as follows: the average pI was 8.95 (ranging from 4.5 to 10.88), and 94% of proteins had a pI above 7. Therefore, the pI is in the basic range, and the proteins are rich in basic aa; the average Mw = 44.3 kDa (ranging from 6.25 to 181.45 kDa). The Mw and pI data in Group I and Group III were more scattered, and those in Group II and Group IV were more concentrated (Figure 3A); the average instability index was = 46.7 (ranging from 30.19 to 66.65); the average aliphatic index was = 76.0 (ranging from 61.97 to 119.25); and the average GRAVY was = −0.21 (ranging from −0.667 to 0.346). Thus, DUF1005 is a hydrophilic protein except for some GRAVY > 0 in gymnosperms. The instability index values indicated that DUF1005 was relatively unstable. The subcellular localization prediction results for all 303 DUF1005 proteins indicated that 184 proteins were localized in the chloroplast. Moreover, from Group I to Group IV, the number of aa and the molecular weight continued to increase, and the aliphatic index continued to decrease. Overall, these results indicated that DUF1005 family members were unstable and hydrophilic proteins that were predicted to be located mainly in the chloroplast.

### The Gene Structure and Conserved Motifs in DUF1005 Family Members

To study the structure of DUF1005 genes, we analyzed their DNA sequences and determined the composition of their introns and exons. The GSDS 2.0 software package was used to map the intron–exon structure of the DUF1005 gene family. The results showed that most of the DUF1005 genes contained two exons (Supplementary Figure S2). Among them, Group I was shown to contain two exons (accounting for 70% of the total number of genes in the subfamily), Group II (65%), Group III (72%), and Group IV (88%). However, Group II contained three exons (20%). The DUF1005 domains of GSMUA_Achr4P08250_001 in Group II had nine exons. Slightly more than half the gymnosperms in Group I genes had three to four exons and had very long introns. The gene structure of the DUF1005 gene family was found to be highly conserved among the various subfamilies, and the number and location of exons were similar among the DUF1005 genes in each subfamily, indicating similar function.

Analyzing motif-representing features, such as DNA-binding sites and protein interaction domains, could help us understand common features of gene family sequences and identify any new conserved motif compositions that might not be recorded in public databases. A total of 6 distinct conserved motifs were found, and the sequence logos for the conserved motifs and their distribution in each protein are illustrated in Figure 3B and Supplementary Figure S3. Most of those containing only motif 3 were located in Group I in gymnosperms. Motif 5 was present in only 261 DUF proteins. Motif 5 was distributed at the N-terminus, and motif 2 was distributed at the C-terminus. Other motifs were found in the middle of the domain. Motif 1 and motif 6 were located nearby, and motifs 3 and 4 stayed close together. Investigations of the amino acid composition indicated that the most frequently occurring amino acid in motifs 3 and 2 were S (Ser) and A (Ala), respectively. Then, these motifs were subjected to SMART online server (http://smart.embl-heidelberg.de/smart/change_mode.pl) for annotation, and the results showed that motif 2 was similar to the DSS1_SEM1 domain (PF05160); both had highly conserved C-T-E-D-A-A-A-F-V-A-L-A-A-A-V-D-L-S amino acid sequences. Furthermore, they were found to be associated with proteasome assembly.

### Molecular Cloning of a DUF1005 Gene From Caragana intermedia

**FIGURE 4:**
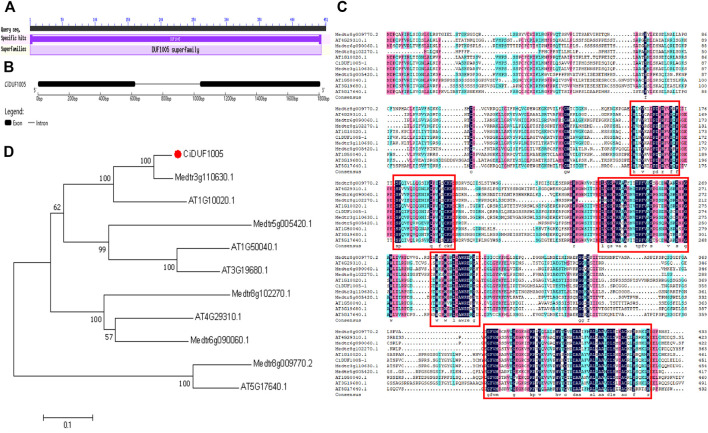
Bioinformatics analysis of DUF1005 in plants. **(A)** The domain of CiDUF1005. **(B)** The gene structure of CiDUF1005. **(C)** Multiple sequence alignment of CiDUF1005 and its homologs. The colors of the conserved residues represent different levels of conservation of each column in the alignment. The five basically conserved areas are represented by red rectangles. **(D)** Phylogenetic tree analysis of CiDUF1005 and its homologs.

We identified a sequence containing the DUF1005 domain from the drought-treated transcriptome database (SRA accession number: SRP121096) of *C. intermedia* and named it *CiDUF1005*. The amplified cDNA and genomic sequence of *CiDUF1005* were submitted to GenBank (accession number: MZ074325). The full-length cDNA sequence of *CiDUF1005* consisted of 1,399 nucleotides and contained a 1,356-bp open reading frame (ORF). The polypeptide deduced by *CiDUF1005* has 451 amino acid residues, and it contains a conserved CiDUF1005 domain with 444 amino acids (Figure 4A). The DUF1005 protein has a predicted theoretical molecular mass of 48.4 kDa and an isoelectric point of 9.25, which were calculated using the ExPASy-Bioinformatics Resource Portal. Comparing the nucleotide sequence of the 1,808-bp genomic DNA with the cloned cDNA showed that *CiDUF1005* comprised one intron and two exons, the first exon was 623 bp, the intron was 415 bp, and the second exon was 670 bp in length (Figure 4B).

The protein sequences of DUF1005 with its homologs from *Arabidopsis* and *Medicago truncatula* were compared (Figure 4C). The results showed that the sequence was largely conserved between species and had high homology. Phylogenetic tree analysis showed that CiDUF1005 was closely related to AT1G10020 and Medtr3g110630.1 (Figure 4D). Multiple sequence alignment of protein sequences from three species was performed. Basically, there were 5 primarily conserved regions with high similarity, which is colored in black. In addition, several secondary and tertiary levels of conservation were detected and are colored pink or blue. It is noticeable that the DUF1005 domain covers most of the amino acid residues in all the DUF1005 proteins.

### Expression Patterns of CiDUF1005

**FIGURE 5:**
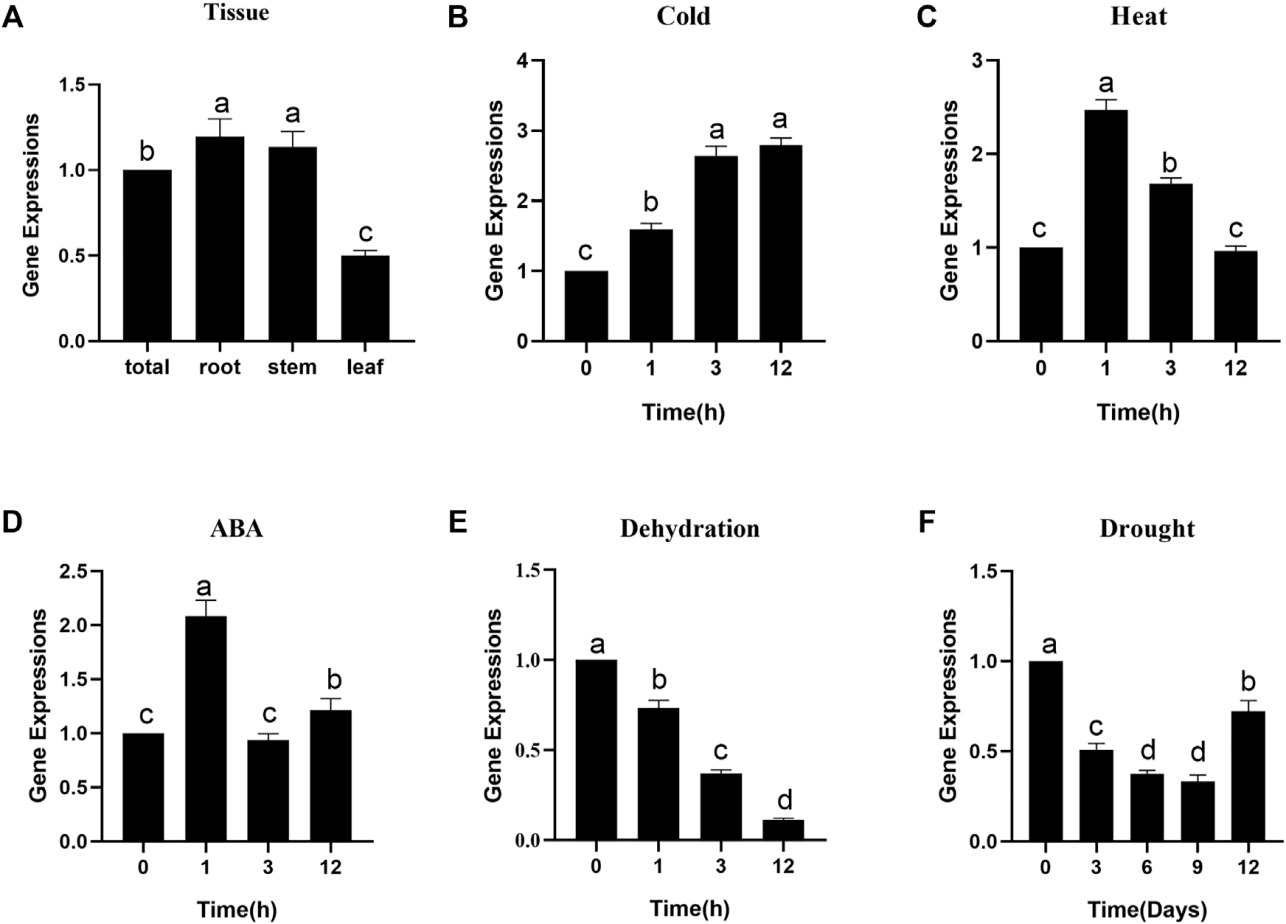
The expression pattern of *CiDUF1005* in *Caragana intermedia*. **(A)** The expression of *CiDUF1005* roots, stems, leaves, and whole seedlings (total). **(B–F)** Pattern of CiDUF1005 expression under different treatments, such as 4°C **(B)**, 42°C **(C)**, and 200 μM of ABA **(D)**, dehydration **(E)**, and drought **(F)**. Different letters indicate significant differences at *p* < 0.05 according to Duncan’s multiple range test.

To gain more insight into the role of *CiDUF1005* in plant growth and development, we used qRT–PCR to examine the transcription level expression of *CiDUF1005* in roots, stems, and leaves (Figure 5). Our results showed that *CiDUF1005* was expressed in all tested tissues, but there were no significant differences. We further examined the expression level of *CiDUF1005* under conditions such as cold, heat, dehydration, and drought treatments, as well as under the hormone abscisic acid (ABA), which is a plant growth regulator and stress hormone (Zhang et al., 2006; Lata and Prasad, 2011). Our results showed that the transcript of *CiDUF1005* could be upregulated within 1 h after cold treatment, reaching a maximum at 12 h. For heat stress, its transcript increased to the maximum level within 1 h and then decreased. Similarly, ABA treatment also rapidly induced a 2-fold increase, followed by a decrease to the basal level. Surprisingly, the expression of *CiDUF1005* decreased with both dehydration and drought treatments, and its expression level was reduced by 10-fold under dehydration. The transcript of *CiDUF1005* started to decrease continuously after 3 days of drought treatment. This was consistent with the *CiDUF1005* trend in the drought-treated transcriptome database (Supplementary Figure S4). Collectively, these results suggested that *CiDUF1005* was regulated by abiotic stresses.

### *CiDUF1005* Regulated Primary Root Elongation and Enhanced Lateral Root Formation

**FIGURE 6:**
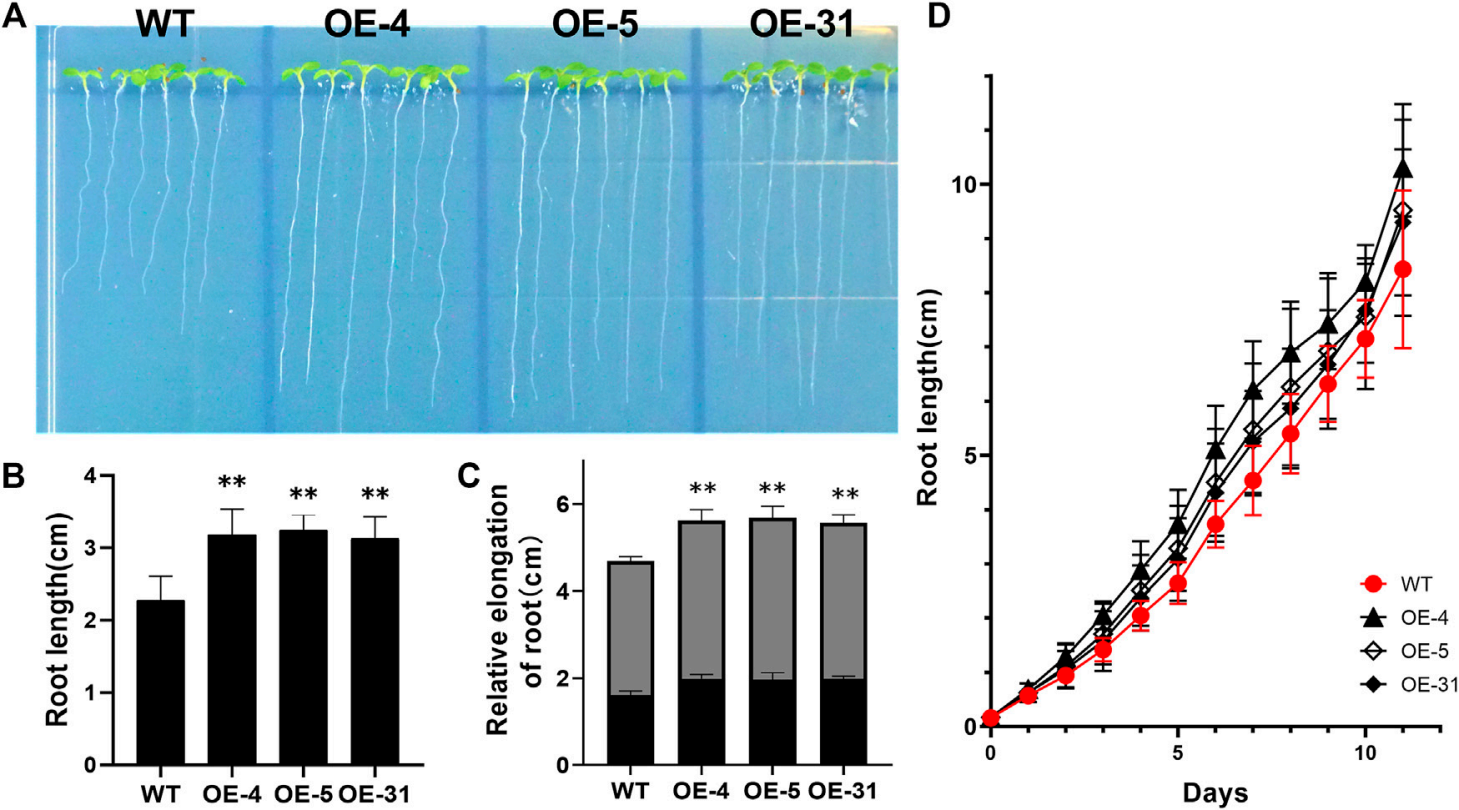
Phenotypic differences in the primary root length between the wild type and OE lines. **(A)** Root morphology of the OE seedlings and wild type. Five-day-old seedlings cultured in 1/2 Murashige and Skoog (MS) were observed. **(B)** The primary root length of 5-day-old wild type and the transgenic seedlings. **(C)** The relative elongation of roots after the third (black) and fifth (gray) days. *N* = 36 ± SD from three independent biological replicates. **(D)** The primary root length comparison between the OE lines and wild type during 11 days of growth. The error bars represent the means of three technical replicates ±SD. Statistically significant differences from the control group are indicated as **p < 0.01.

To investigate the function of CiDUF1005, we generated homozygous transgenic *Arabidopsis* with overexpressed *CiDUF1005*. Among the 7 homozygous transgenic lines, 3 lines with the highest *CiDUF1005* expression level were selected for further analysis and were designated OE-4, OE-5, and OE-31 (Supplementary Figure S5). Although no obvious phenotypes between the WT and OE lines were observed during the mature stage, there were significant differences at the seedling stage. In 5-day-old seedlings, the primary roots were much longer in the OE lines than in the WT when grown on 1/2 MS medium (Figures 6A,B). We also used an empty vector transformed *Arabidopsis* line as a control for comparison with the OE lines, and the results were consistent with the use of the WT, which was used as a control. The primary roots were much longer in the OE lines than in the control line (Supplementary Figure S6). To rule out the possibility that the difference in primary root length was caused by the difference during germination, the growth rate of roots of the OE seedling from the third day to the fifth day was analyzed, and the results showed that the root growth rate of the OE lines increased compared with that of the WT (Figure 6C). The relative rates of the primary root elongation of the three OE lines were 1.4-, 1.43-, and 1.37-fold compared to those of the WT. The evidence suggested that overexpressing *CiDUF1005* caused the primary root to grow significantly faster under normal growth conditions.

**FIGURE 7:**
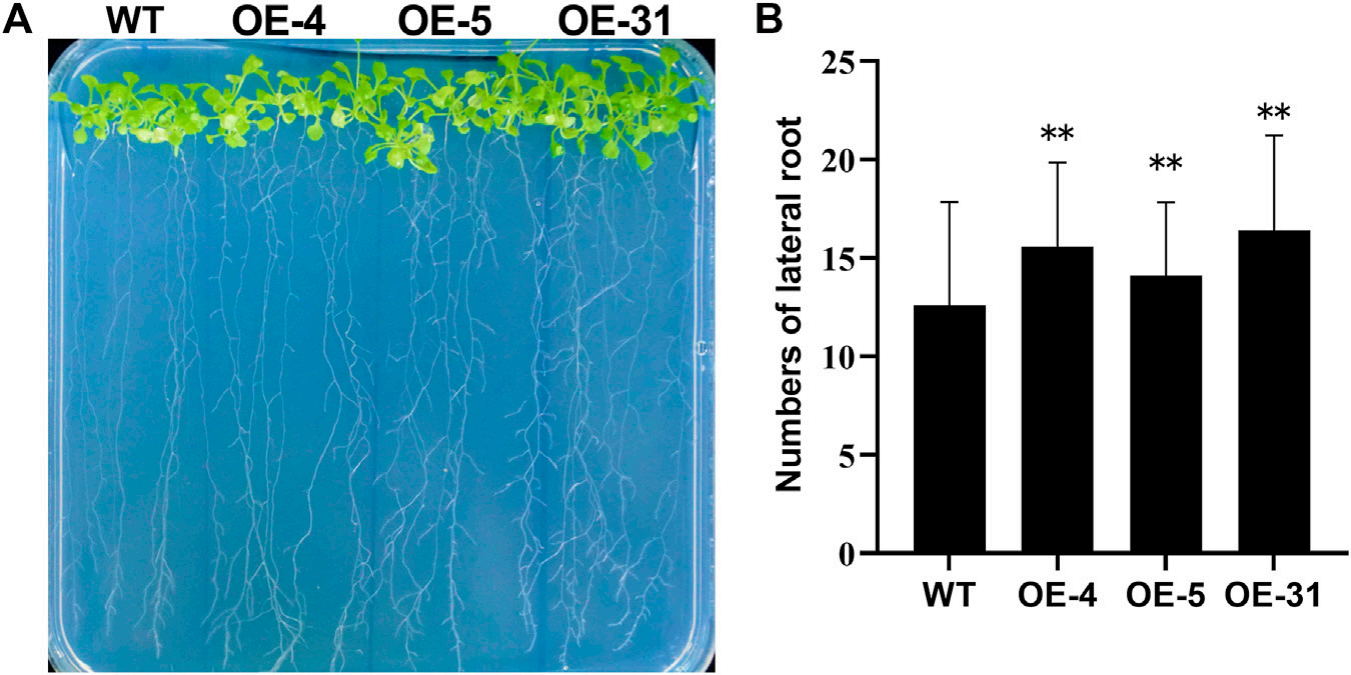
Phenotypic differences in the number of lateral roots between the wild type and OE lines. **(A)** The lateral root morphology of the OE lines and wild-type seedlings. Eleven day-old seedlings cultured in 1/2 Murashige and Skoog (MS) were observed. **(B)** The number of lateral roots of the 11-day-old wild type and the OE lines. *N* = 36 ± SD from three independent biological replicates. The error bars represent the means of three technical replicates ±SD. Statistically significant differences are indicated as **p < 0.01.

To determine at which period *CiDUF1005* overexpression promoted root elongation, we measured the root length of the seedling from the first day to the 11th day (Figure 6D and Supplementary Figure S7) after germination. The length of the primary root of the OE lines was longer than that of the WT from the third day. The difference was the largest during the period from the fifth day to the ninth day. After 11 days of growth, the difference in root length decreased gradually between the OE lines and WT (Supplementary Figure S7), while there was no significant difference in adult plants. We also noticed that the OE lines displayed an almost 1.3-fold increase in the number of lateral roots on the 11th day (Figures 7A,B).

To further study the responses of *CiDUF1005* that were induced by ABA during root development, we measured the root length of *CiDUF1005* under ABA treatment. In response to the increasing ABA concentration, the relative elongation of the primary root of OE lines was still longer than that of the WT (Supplementary Figure S8). Additionally, to further investigate the response of *CiDUF1005* to drought stress, the primary roots of the WT and OE lines were treated with 350 mM of mannitol. After 5 days of mannitol stress, the OE lines had a longer root length than the WT (Supplementary Figures S9A,B). However, there was no significant difference in the primary root length between the WT and OE lines when measured at day 11 (Supplementary Figures S9C,D).

Taken together, these data strongly confirmed that transgenic *CiDUF1005* expression increased the number of lateral roots and promoted primary root elongation during the seedling period.

### Expression Levels of Some Related Root Development Genes

**FIGURE 8:**
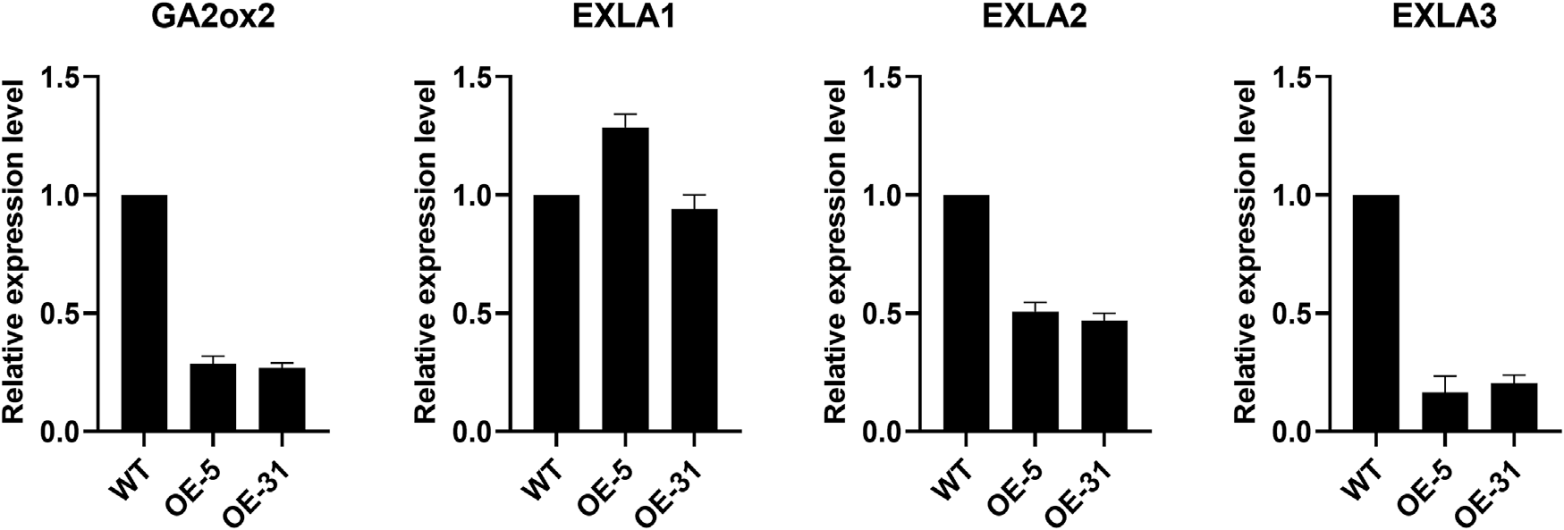
Expression levels of the root development genes. Relative expression levels of *GA2ox2*, *EXLA1 EXLA2*, and *EXLA3* in 5-day-old seedlings. The error bars represent the means of three technical replicates ±SD.

To understand how *CiDUF1005* regulates root development in *Arabidopsis*, we investigated the expression of ABA-related genes (*ARR5*, *ABF1*, *ABF2*, *COR15B*, *RD29B*, *RD26*, *ABI1*, *ABI5*, and *RAB18*) in OE lines and the WT. In contrast, no clear differences in their expression levels between the WT and OE lines were detected (Supplementary Figure S10). Eventually, we searched the literature and looked for genes that were related to root development in the WT and OE lines. These include *GIBBERELLIN 2-OXIDASE2* (*GA2ox2*) (Rieu et al., 2008; Ubeda-Tomás et al., 2008; Moubayidin et al., 2010) and *Expansin-like A* (*EXLA*) (Cosgrove, 2000). The results showed the transcript levels of *GA2ox2* (*AT1G30040*), *EXLA1* (*AT3G45970*), *EXLA2* (*AT4G38400*), and *EXLA3* (*AT3G45960*). *GA2ox2*, *EXLA2*, and *EXLA3* were significantly lower in the transgenic lines than in the WT (Figure 8). However, there was no difference in the transcript levels of *EXLA1* between the WT and transgenic plants. This result suggests that *CiDUF1005* might influence primary root elongation by regulating the expression of *GA2ox2*, *EXLA2*, and *EXLA3*, by a currently unknown mechanism.

## Discussion

The rapid growth of biological sequence databases has accompanied the development of high-throughput genomic and sequencing techniques (Mudgal et al., 2015). In addition, the fraction of DUF gene families in Pfam entries has increased over the past decade (Finn et al., 2008). Since the last release of Pfam 34.0, it has built 935 new families, killed 15 families, and created 11 new clans. UniProt Reference Proteomes have increased by 21% since Pfam 33.1 and now contain 47 million sequences. The identification of the biological functions of a large number of DUF family genes is a huge challenge. Many studies have shown that genome-wide identification and expression analysis could help researchers understand the origin, diversity, and biological functions of these DUF gene families (Moturu et al., 2018).

As a gene family that encodes proteins with unknown functions, DUF1005 appears to be a plant-specific domain (Batelli et al., 2012). In the present study, we identified 302 DUF1005 family members from 58 different plant species (Figure 1). Most species had 3–9 DUF1005 genes. Although plants were exposed to different environments during evolution, no gene differentiation of DUF1005 occurred, and no special expansion occurred in our analysis. The selected plant species in this study had different genome sizes and different rounds of whole-genome duplication, but they had a similar number of DUF1005. This result indicated that most of the genes in the DUF1005 family were not eliminated by environmental selection; instead, they showed high conservation during evolution. The finding of similar conserved motifs within the DUF1005 family further supports this point. This demonstrates that the domains of all DUF1005 family members support this notion. They remain to be studied in detail from an evolutionary perspective. We did not identify any DUF1005 members in algae strains, indicating that this gene family might originate from the landing of plants (Supplementary Table S3). In addition, no obvious expansion of this family occurred from early plant bryophytes and lycophytes to relatively late-diverging gymnosperms and angiosperms (Supplementary Table S1).

Phylogenetic analysis revealed that 303 DUF1005 were separated into four groups, and their diversification during evolution was revealed (Figure 2 and Supplementary Figure S1). Among these, Group I covered almost all the plant species, Groups II to IV were specific to the angiosperms, and Group IV was dicotyledon-specific. Although the protein sequences of DUF1005 varied greatly in different species and shared low overall sequence similarity, they possessed one highly conserved region (Figure 4).

The DUF1005 gene family in *Arabidopsis* is composed of 5 genes. The biological function of most *DUF1005*-encoded proteins remains unknown, which is partly because very few phenotypes related to this family have been uncovered thus far. Among the limited *DUF1005* genes with established functions, *AT5G17640*, also known as *Asg1*, belonged to the earliest group I. Although *Asg1* had no sequence similarities that could suggest its biological function in plant cells, its isolation through functional screening indicated that *Asg1* was induced by salt stress in both *Solanum tuberosum* and *Arabidopsis* and by ABA in *Arabidopsis* (Batelli et al., 2012).

Expression analysis showed that *CiDUF005* was regulated by various abiotic stresses (Figure 5). To study the abiotic stress response in the OE lines of *CiDUF1005*, WT and seedlings of the OE lines were subjected to mannitol stress. After 5 days of 350 mM of mannitol stress, except for the significance “**” of OE-4, the OE-5 significance “**” was reduced to “*,” and OE-31 was not significant compared to the untreated condition (Supplementary Figures S9A,B). This result was consistent with previous findings that the expression of *CiDUF1005* was decreased under drought treatment (Figure 3 and Supplementary Figure S4). The accumulation of ABA in the root apex was necessary for maintaining elongation in the apical region of the elongation zone (Saab et al., 1992). Further tests are needed to confirm whether the primary root elongation of *CiDUF1005* was regulated by ABA signaling, while the OE lines still had longer primary roots than the WT after ABA treatment (Supplementary Figure S8), suggesting that CiDUF1005 was not directly involved in the ABA pathway.

The root system of plants is a primary determinant of growth potential. A root trait contains many factors, such as root length, number, diameter, and root configuration in the soil profile. Root length is one of the most important parameters and primarily reflects a plant’s ability to acquire nutrients from the soil (Teo et al., 1995). However, the underlying mechanisms through which DUF1005 controls root development remain elusive.

Previous studies have shown that DUF1005 might be associated with plant development; for example, *VrDUF1005* plays a role in regulating the shape and size of the cell during plant growth (Yun et al., 2020). In our study, CiDUF1005 regulated primary root elongation (Figure 6) and enhanced lateral root formation (Figure 7) during the plant seedling stage.

Therefore, we searched the literature and looked for genes that were related to root development. *GA2ox2*, which encodes an oxidase that inactivates bioactive C19 gibberellins via 2-oxidation, is a major gibberellin (GA) inactivating pathway in *Arabidopsis*, and GA delays the switch from cell division to expansion in *Arabidopsis* roots (Rieu et al., 2008; Ubeda-Tomás et al., 2008; Moubayidin et al., 2010). *GA2ox2* expression controls *Arabidopsis* root meristem cell number, and *GA2ox2*OE lines formed shorter roots than those of the WT (Li et al., 2017).

Expansin loosened plant cell walls and was involved in cell enlargement and various abiotic stress responses. EXLA is a member of the plant extended protein superfamily. Three expansin-like genes were found in *Arabidopsis*: *EXLA1*, *EXLA2*, and *EXLA3* (Cosgrove, 2000). Functional studies have shown that expansins are involved in many developmental processes in plants. Previous research indicated that the roots of *exla2* mutants were longer (123%) than the WT roots (Abuqamar et al., 2013). This was consistent with our earlier study, which showed that the transcript levels of *EXLA2* were significantly lower in the transgenic plants than in the WT plants (Figure 8). We speculated that CiDUF1005 might regulate root development by influencing the expression of *EXLA2* and *GA2ox2*.

Root length is determined by cell division in the root meristem and cell elongation in the root elongation zone (Beemster et al., 2003). Cell proliferation and division are largely related to mitotic cyclin-dependent kinase activity, such as CCS52A (Vanstraelen et al., 2009). Expansion of root cells in the elongation zone contributes to primary root growth and is largely regulated by plant hormones. It is well established that plant hormones play a key role in root development (Péret et al., 2009). The major plant hormones—ABA, brassinosteroids, cytokinin, ethylene, GA, and auxin (IAA)—are all key regulators of primary root growth (Petricka et al., 2012). Therefore, the detection of hormone content in *CiDUF1005* transgenic lines should be helpful to confirm whether DUF1005 regulates root development through hormone signaling pathways, and this will be the main topic in our future research.

## Data Availability

The original contributions presented in the study are included in the article/Supplementary Material, further inquiries can be directed to the corresponding authors.
