# Supplementary figures and images for: DUF1005 Family Identification, Evolution Analysis in Plants, and Primary Root Elongation Regulation of CiDUF1005 From Caragana intermedia
Source: Front Genet. 2022 Mar 29;13:807293. doi: 10.3389/fgene.2022.807293 (PMC9001952; doi:10.3389/fgene.2022.807293)

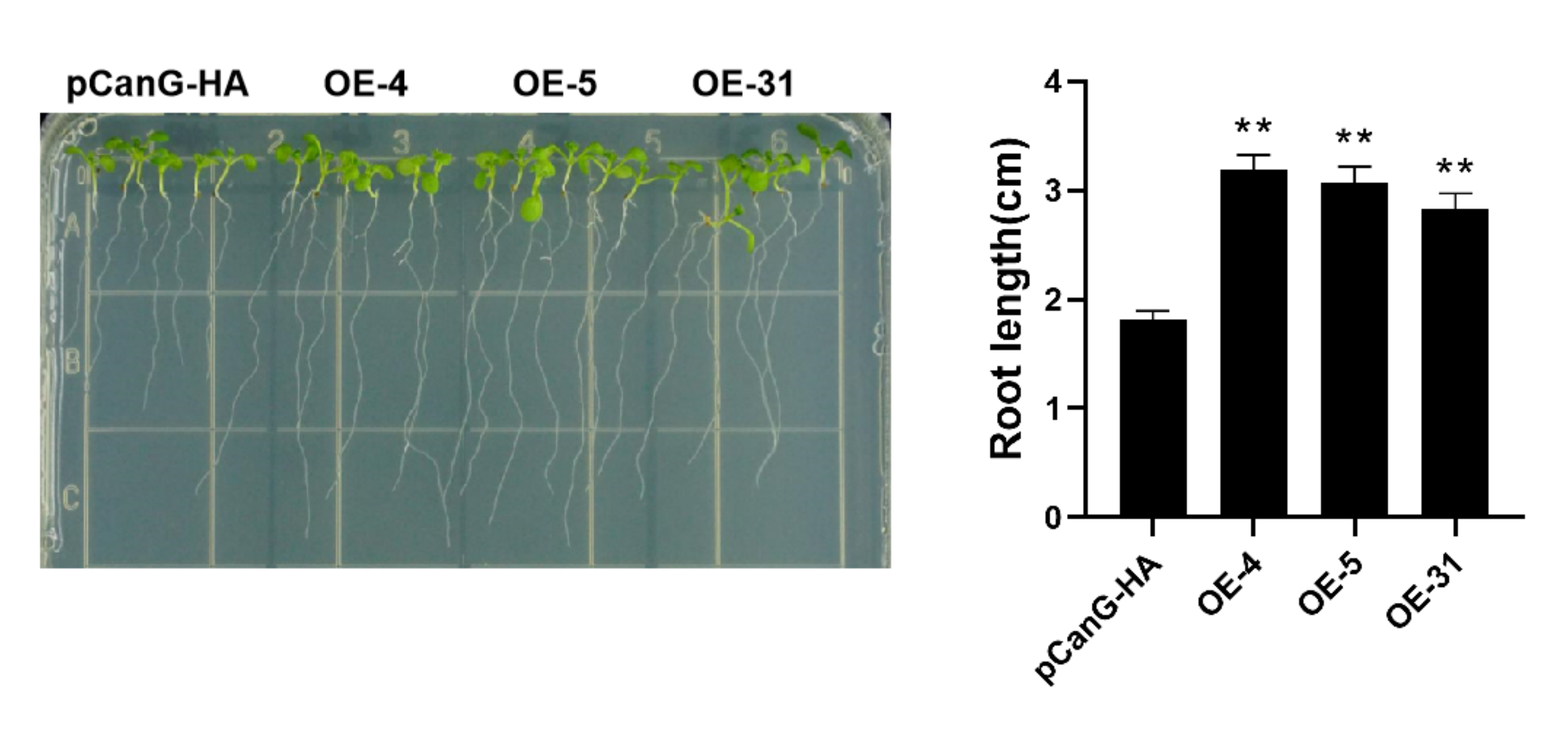

Supplement: Supplementary file 3 [file Image6.TIF]

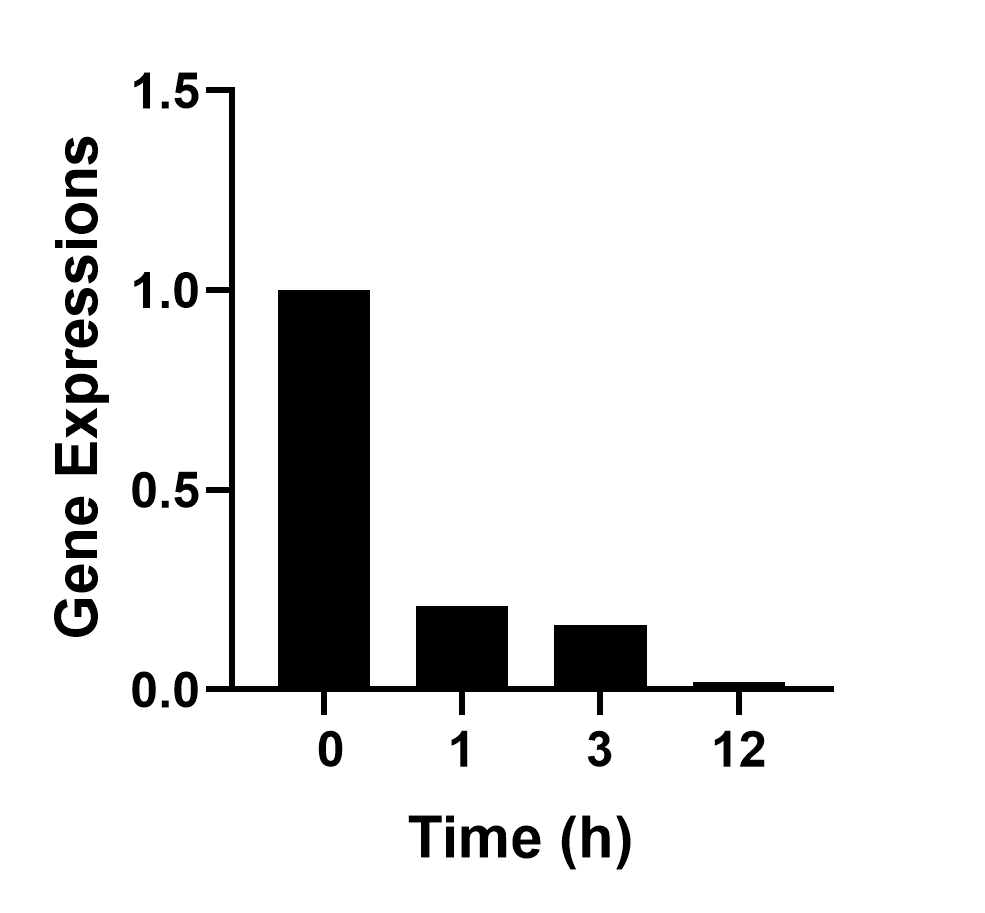

Supplement: Supplementary file 6 [file Image4.TIF]

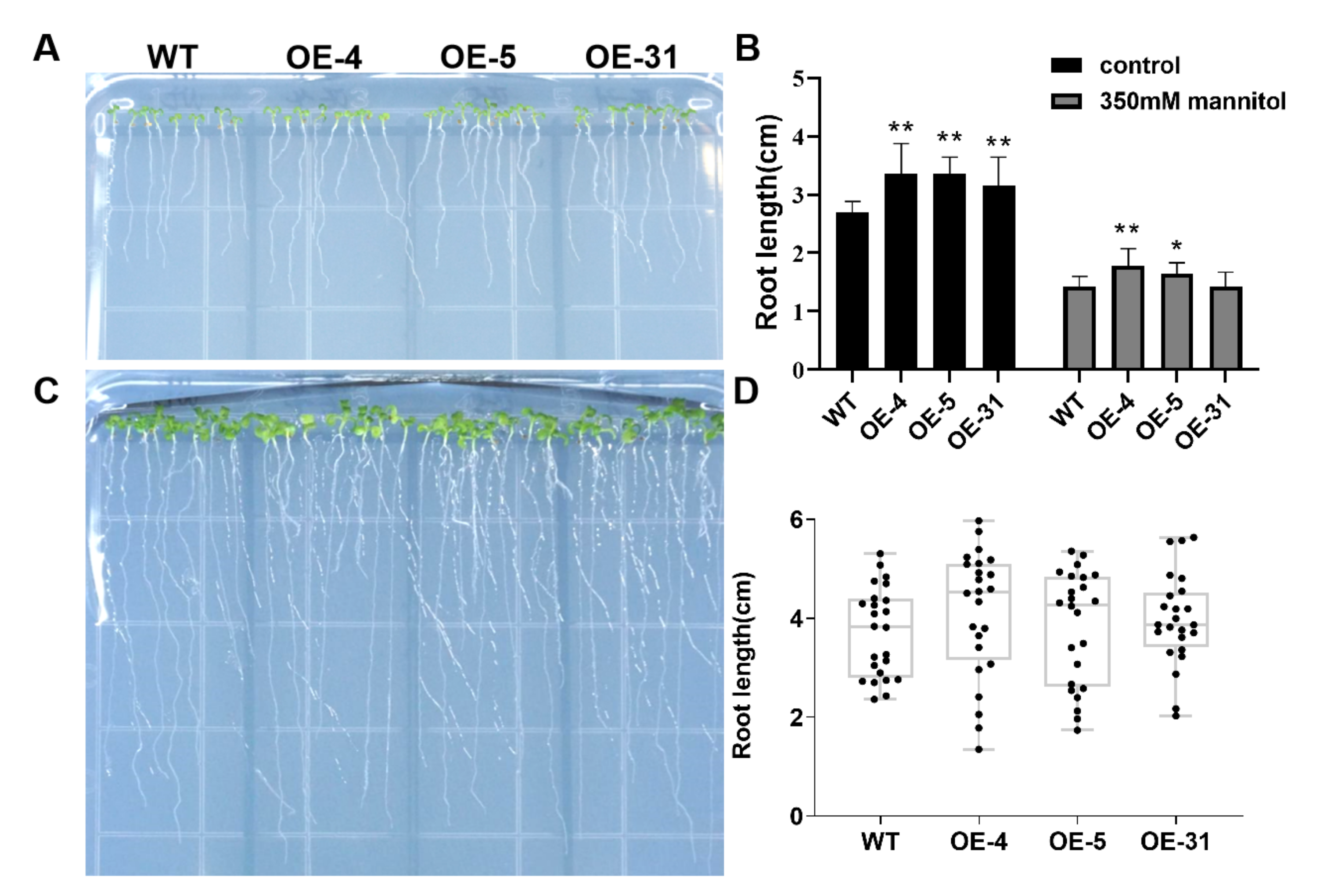

Supplement: Supplementary file 7 [file Image9.TIF]

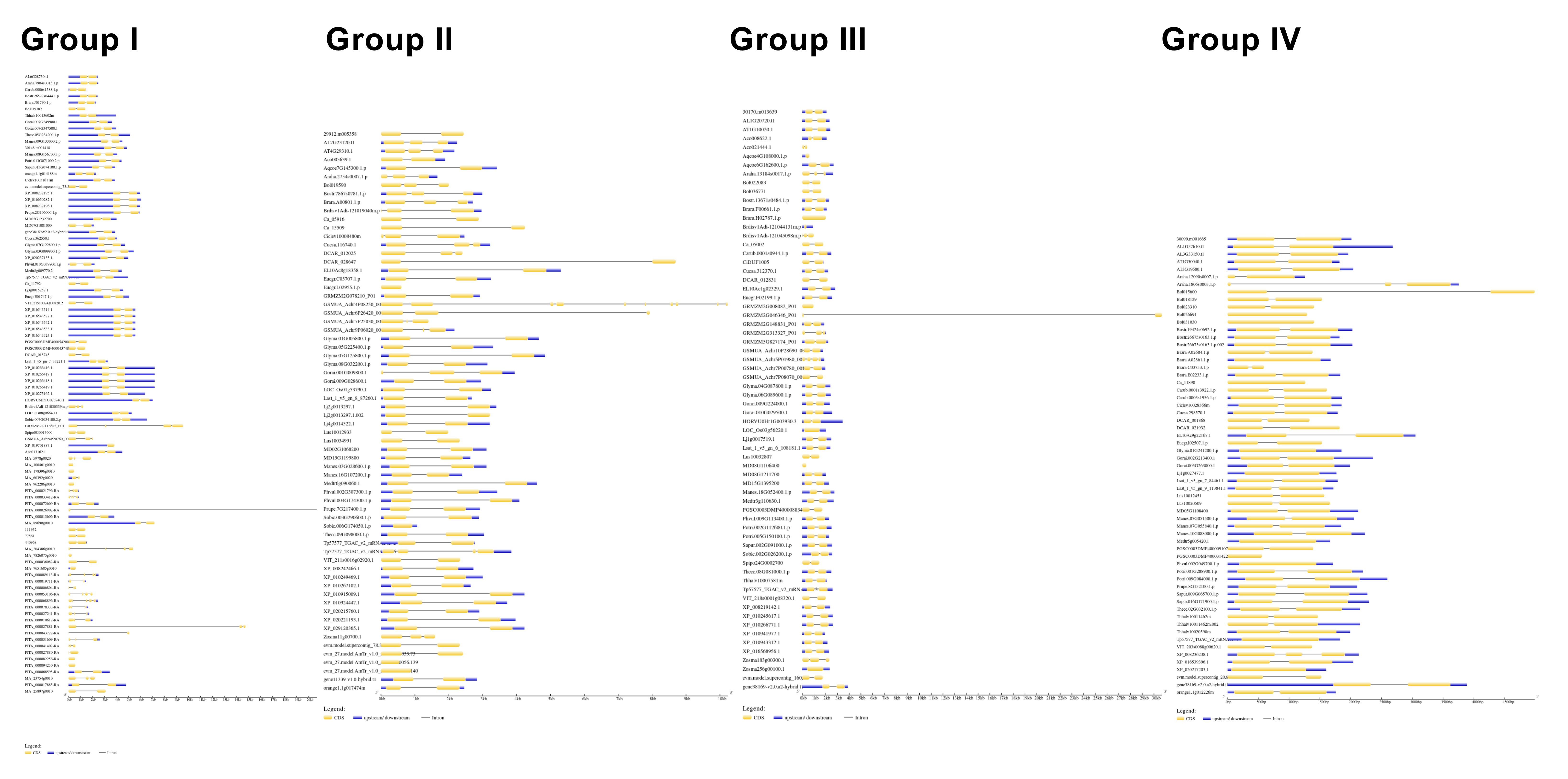

Supplement: Supplementary file 8 [file Image2.TIF]

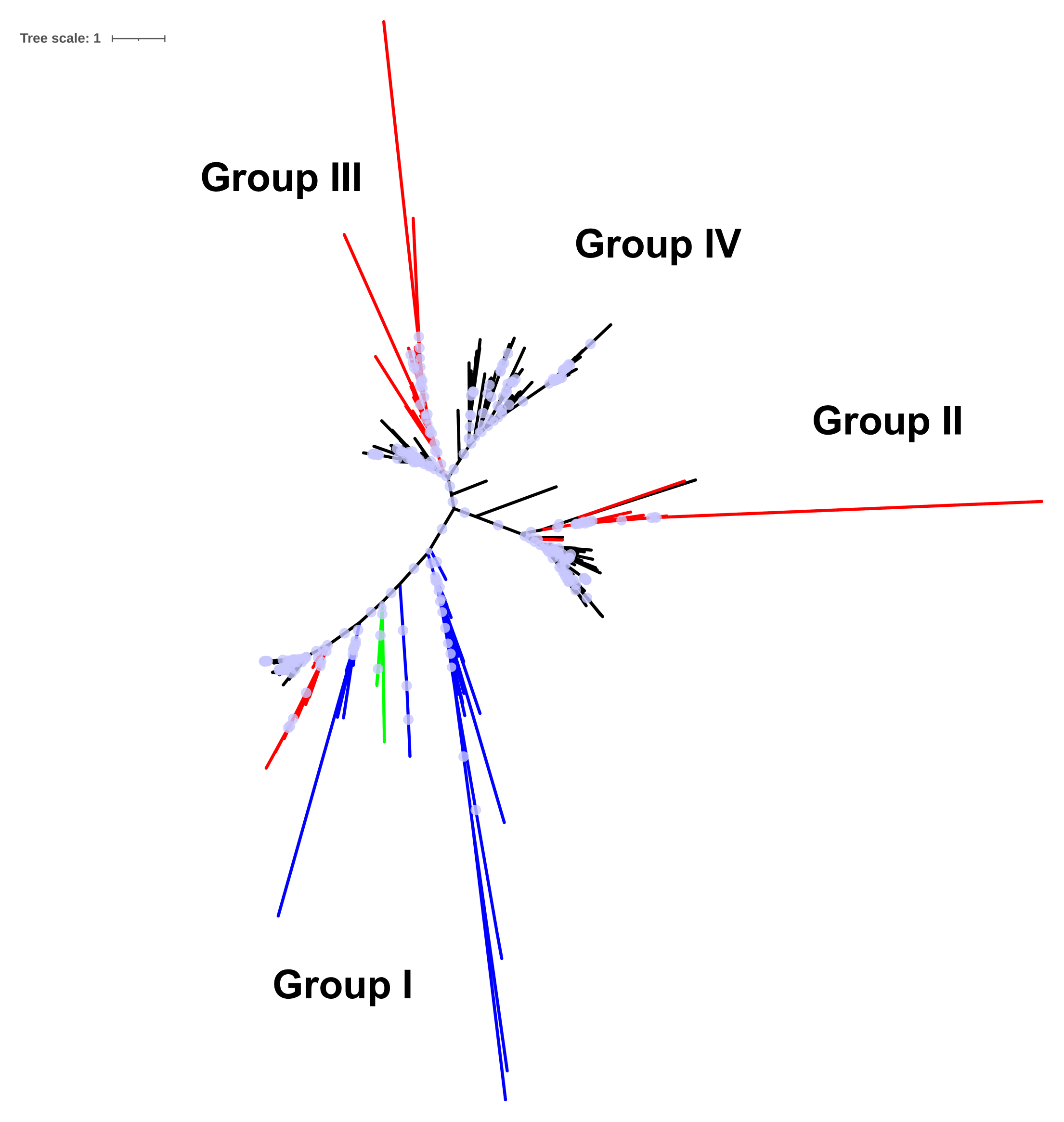

Supplement: Supplementary file 9 [file Image1.TIF]

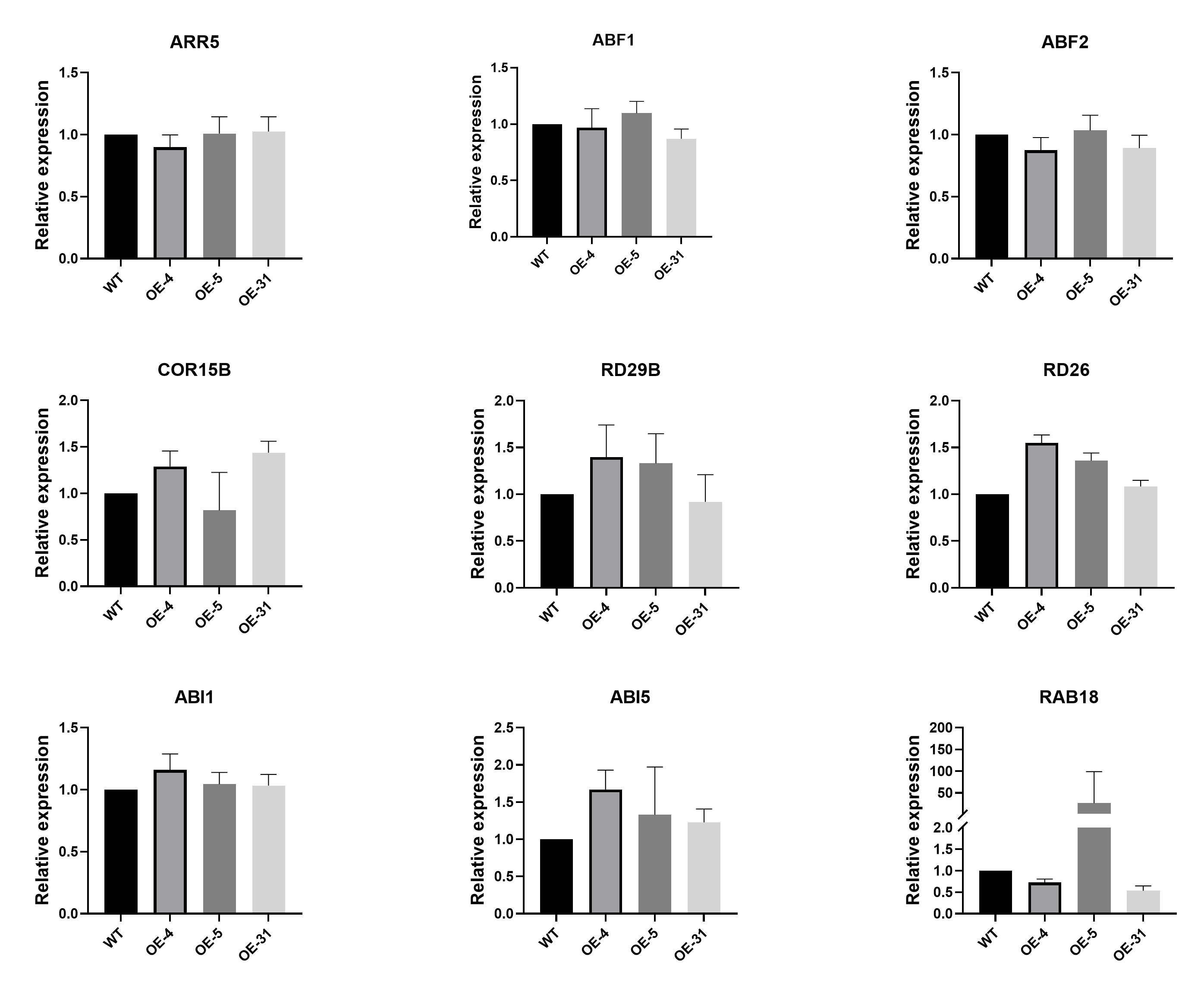

Supplement: Supplementary file 10 [file Image10.TIF]

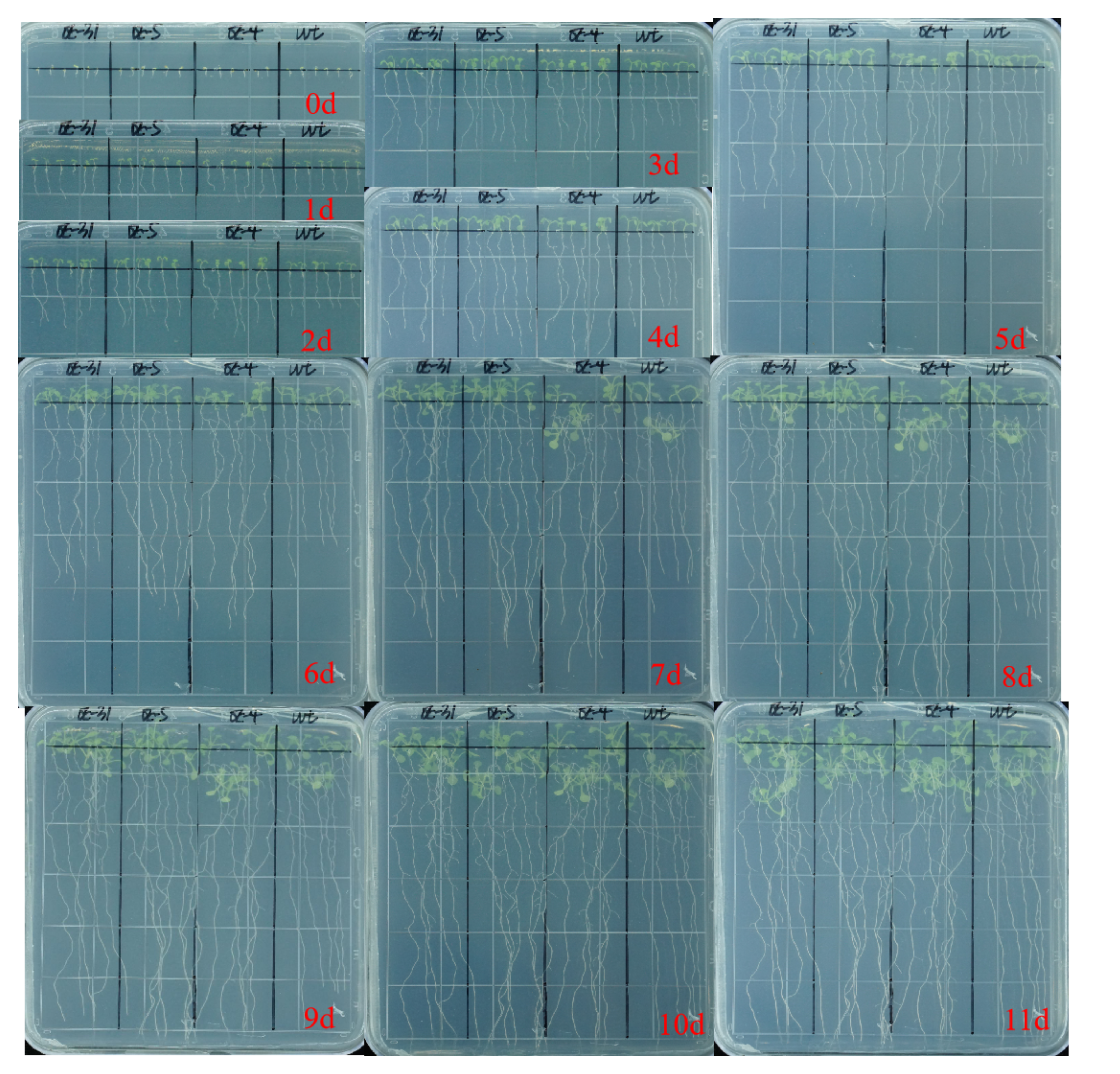

Supplement: Supplementary file 11 [file Image7.TIF]

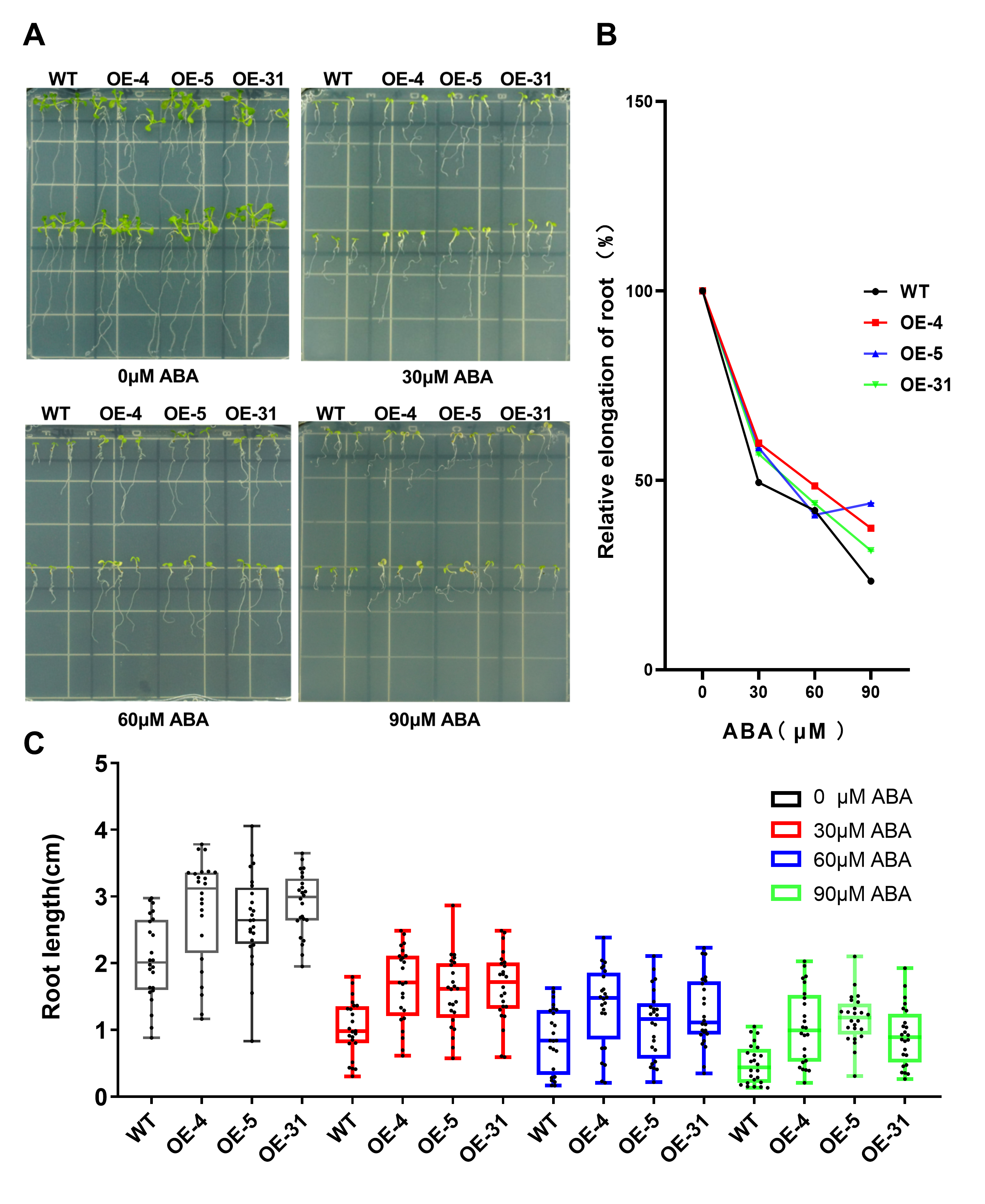

Supplement: Supplementary file 14 [file Image8.TIF]

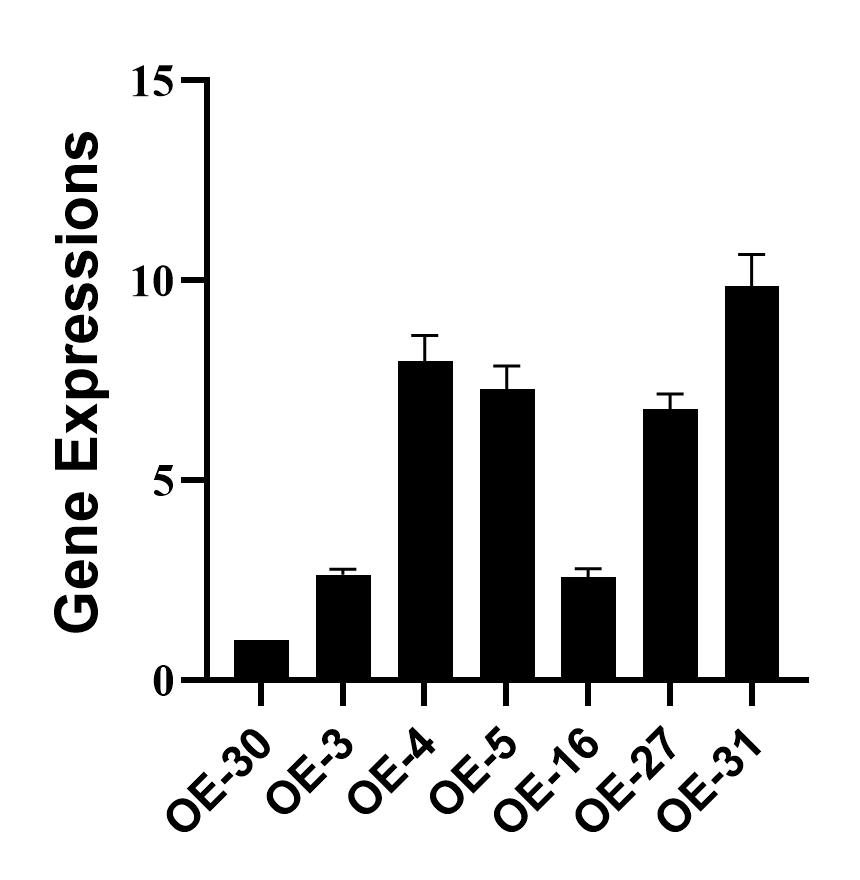

Supplement: Supplementary file 15 [file Image5.TIF]
