# Supplementary material for: DUF1005 Family Identification, Evolution Analysis in Plants, and Primary Root Elongation Regulation of CiDUF1005 From Caragana intermedia
Source: Front Genet. 2022 Mar 29;13:807293. doi: 10.3389/fgene.2022.807293 (PMC9001952; doi:10.3389/fgene.2022.807293)
Supplement: Supplementary file 4 [file Table1.DOCX]

**Table S1** Information on the species and their genomes used for *DUF1005* identification.

| Lineage | Organism | Genome size | Number of predicted genes |
| --- | --- | --- | --- |
| Alage | *Volvox carteri* | 131.2 Mb | 14,247 |
|  | *Chlamydomonas reinhardtii* | 111.1 Mb | 17,741 |
|  | *Ostreococcus lucimarinus* | 13.2Rh | 7794 |
|  | *Micromonas pusilla* | 22Mb | 10660 |
| Moss | *Physcomitrella patens* | 473Mb | 32,926 |
| Lycophytes | *Selaginella moellendorffii* | 212.5 Mb | 22,273 |
| Gymnosperms | *Picea abies* | 19.6Gb | 91,045 |
|  | *Pinus taeda* | 22 Gb | 66,632 |
| Basal angiosperms | *Amborella trichopoda* | 706 Mb | 26,846 |
| Monocots | *Oryza sativa* | 372 Mb | 42,189 |
|  | *Brachypodium distachyon* | 272 Mb | 36,647 |
|  | *Zea mays* | 2300 Mb | 40,557 |
|  | *Ananas comosus* | 382Mb | 27,024 |
|  | *Hordeum vulgare* | 4.6Gb | 39,734 |
|  | *Sorghum bicolor* | 732.2 Mb | 34,129 |
|  | *Elaeis guineensis* | 1.8 Gb | 34,802 |
|  | *Musa acuminata* | 472 Mb | 36,528 |
|  | *Spirodela polyrhiza* | 158-Mb | 19,623 |
|  | *Zostera marina* | 202.3 Mb | 20,450 |
| Dicots | *Eucalyptus grandis* | 691 Mb | 36,349 |
|  | *Populus trichocarpa* | 422.9 Mb | 42,950 |
|  | *Medicago truncatula* | 390 Mb | 62,319 |
|  | *Arabidopsis thaliana* | 135 Mb | 27,416 |
|  | *Aquilegia coerulea* | 306.5 Mb | 30,023 |
|  | *Phaseolus vulgaris* | 537.2 Mb | 27,433 |
|  | *Glycine max* | 978.5Mb | 56,044 |
|  | *Trifolium pratense* | 309 Mb | 40,868 |
|  | *Cajanus cajan* | 858 Mb | 59,515 |
|  | *Lotus japonicus* | 517.54 Mb | 28,251 |
|  | *Cicer arietinum* | 738.09 Mb | 28,269 |
|  | *Prunus mume* | 280 Mb | 31,390 |
|  | *Prunus persica* | 224.6 Mb | 26,873 |
|  | *Malus domestica* | 881.3 Mb | 63,514 |
|  | *Fragaria vesca* | 240Mb | 32,831 |
|  | *Capsella rubella* | 134.8 Mb | 26,521 |
|  | *Boechera stricta* | 196.5 Mb | 27,416 |
|  | *Arabidopsis lyrata* | 207Mb | 33,132 |
|  | *Eutrema salsugineum* | 243.1 Mb | 26,351 |
|  | *Arabidopsis halleri* | 145.5 Mb | 25,008 |
|  | *Brassica oleracea* | 630 Mb | 35,400 |
|  | *Brassica rapa* | 283.8 Mb | 40,492 |
|  | *Cucumis sativus* | 203Mb | 21491 |
|  | *Daucus carota* | 421 Mb | 32,113 |
|  | *Carica papaya* | 135Mb | 27769 |
|  | *Gossypium raimondii* | 737.8 Mb | 37,505 |
|  | *Theobroma cacao* | 346 Mb | 29,452 |
|  | *Ricinus communis* | 400 Mb | 31,237 |
|  | *Manihot esculenta* | 582.25 Mb | 33,033 |
|  | *Solanum tuberosum* | 723Mb | 39,028 |
|  | *Capsicum annuum* | 3.3Gb | 35,336 |
|  | *Citrus sinensis* | 319 Mb | 25,376 |
|  | *Citrus clementina* | 301.4 Mb | 24,533 |
|  | *Linum usitatissimum* | 318.3 Mb | 43,471 |
|  | *Lactuca sativa* | 2.7 Gb | 38,910 |
|  | *Vitis vinifera* | 487Mb | 26346 |
|  | *Beta vulgaris* | 628 Mb | 24,255 |
|  | *Nelumbo nucifera* | 929 Mb | 26,685 |
|  | *Salix purpurea* | 392.0 Mb | 37,865 |

The data were obtained from the Phytozome 12, ConGenIE and NCBI..

*Volvox carteri v2.1, Chlamydomonas reinhardtii v5.5, Ostreococcus lucimarinus v2.0, Micromonas pusilla CCMP1545 v3.0, Physcomitrella patens v3.3, Selaginella moellendorffii* *v1.0, Picea abies v1.0, Pinus taeda v1.0, Amborella trichopoda v1.0, Oryza sativa v7_JGI (Rice), Brachypodium distachyon Adi-12 v1, Zea mays (B73 RefGen_v4), Ananas comosus v3, Hordeum vulgare r1, Sorghum bicolor v3.1.1, Eucalyptus grandis v2.0 (Rose gum), Musa acuminata v1, Spirodela polyrhiza v2, Zostera marina v2.2, Eucalyptus grandis v2.0, Populus trichocarpa v3.1 (Poplar), Medicago truncatula Mt4.0v1 (Barrel medic), Arabidopsis thaliana TAIR10, Aquilegia coerulea v3.1, Phaseolus vulgaris v2.1, Glycine max Wm82.a2.v1, Trifolium pratense v2, Cajanus cajan C.cajan_V1.0, Lotus japonicus Lj1.0v1, Cicer arietinum v1.0, Prunus mume_V1.0, Prunus persica v2.1, Malus domestica v1.1, Fragaria vesca v2.0.a2, Capsella rubella v1.1, Boechera stricta v1.2, Arabidopsis lyrata v2.1, Eutrema salsugineum v1.0, Arabidopsis halleri v1.1, Brassica oleracea capitata v1.0, Brassica rapa FPsc v1.3, Cucumis sativus v1.0, Daucus carota v2.0, Carica papaya ASGPBv0.4, Gossypium raimondii v2.1, Theobroma cacao v1.1, Ricinus communis v0.1, Manihot esculenta v7.1, Solanum tuberosum v4.03, Capsicum annuum Pepper Zunla 1 Ref_v1.0, Citrus sinensis v1.1, Citrus clementina v1.0, Linum usitatissimum v1.0, Lactuca sativa V8, Vitis vinifera v2.1, Beta vulgaris EL10_1.0, Nelumbo nucifera Chinese Lotus 1.1, Salix purpurea v5.1.*
