# Supplementary material for: DUF1005 Family Identification, Evolution Analysis in Plants, and Primary Root Elongation Regulation of CiDUF1005 From Caragana intermedia
Source: Front Genet. 2022 Mar 29;13:807293. doi: 10.3389/fgene.2022.807293 (PMC9001952; doi:10.3389/fgene.2022.807293)
Supplement: Supplementary file 12 [file Table5.DOCX]

**Table S5.** Primers used in this study.

| Name | Usage | Sequence（5’-3’） |
| --- | --- | --- |
| CiDUF1005HA-F | Gene Clone | CGCCTCCCTCGTCGACATGGATCCTTGCCCTTTCGTGCGC |
| CiDUF1005HA-R | Gene Clone | GATCGGGGAAATTCGAGCTCTTCGACAGGGGCAGCAAGATCACGA |
| CiDUF1005qRT-F | Real-time PCR | CAGCGGTGATTATGGATACGGTCTT |
| CiDUF1005qRT-R | Real-time PCR | GCTCACCTCCACCGTAGGCTTG |
| qAtEF1a-F | Real-time PCR | AGAAGGGTGCCAAATGATGAG |
| qAtEF1a-R | Real-time PCR | GGAGGGAGAGAGAAAGTCACAGA |
| qCiEF1a-F | Real-time PCR | CAAAAAGTCCCCTCGTTGTCTC |
| qCiEF1a-R | Real-time PCR | AGCAATCGTTCTTCCTAATGATCTAA |
| GA2ox2-qRT-F | Real-time PCR | GCCTGAGACTAAACCATTATCCG |
| GA2ox2-qRT-R | Real-time PCR | ATTTGAAGACCCGCCGTGT |
| EXLA1-qRT-F | Real-time PCR | GGGTTACATGACAAGAAGCCA |
| EXLA1-qRT-R | Real-time PCR | CATTTTACCGTCGTATCCACC |
| EXLA2-qRT-F | Real-time PCR | CTTGTCCTTAGCAGCAGAGCC |
| EXLA2-qRT-R | Real-time PCR | GGTACAAGAGCTTTATCGCC |
| EXLA3-qRT-F | Real-time PCR | GGCAAACAACAAACAAACACCC |
| EXLA3-qRT-R | Real-time PCR | GGACTCTAGCTTTTAGCATGACCT |
| ARR5-qRT-F | Real-time PCR | CAGAGAACATCTTGCCTCGTATCG |
| ARR5-qRT-R | Real-time PCR | TTAGCTTCAAGCTCTCTCTTGTGCAT |
| COR15B-qRT-F | Real-time PCR | TCTTTCTTGTATTTATTTTCCTCCCAA |
| COR15B-qRT-R | Real-time PCR | CGTATATCAACGACTTCTTGCGCTG |
| RAB18-qRT-F | Real-time PCR | AAAATAATGAGGGTTTGTAACGCAGTC |
| RAB18-qRT-R | Real-time PCR | AAACATCTCAGAACATAAAAAGAGCCAG |
| RD26-qRT-F | Real-time PCR | GTTATTGGAAAGCAACGGGTACTGAC |
| RD26-qRT-R | Real-time PCR | GGCTACGAGAATGTTCTATTAAGCGAT |
| RD29B-qRT-F | Real-time PCR | GAGACAAAACCAAGCACCTACACAG |
| RD29B-qRT-R | Real-time PCR | ACTTTCTGCCCGTAAGCAGTAACA |
| ABI1-qRT-F | Real-time PCR | CGTCTCACATCTTCGTCGCTAACT |
| ABI1-qRT-R | Real-time PCR | TCCACTGAATCACTTTCCCTCCTG |
| ABI5-qRT-F | Real-time PCR | CAAAACCCGTCTAGTGTAATACCCG |
| ABI5-qRT-R | Real-time PCR | CTCCATAGCAAACACCTGCCTGAA |
| ABF1-qRT-F | Real-time PCR | ATGGGAGGAGCAGGGGGTACTG |
| ABF1-qRT-R | Real-time PCR | TCTTCTTCCCCGACCAAACACG |
| ABF2-qRT-F | Real-time PCR | TTATGGAACACAAATGGGTCAGC |
| ABF2-qRT-R | Real-time PCR | GACACAGCACCAACGCCTAAAG |
